# Luting glass ceramic restorations using a selfadhesive resin cement under different dentin conditions

**DOI:** 10.1590/S1678-77572010000300008

**Published:** 2010

**Authors:** Guilherme B. GUARDA, Luciano S. GONÇALVES, Américo B. CORRER, Rafael R. MORAES, Mário A.C. SINHORETI, Lourenço CORRER-SOBRINHO

**Affiliations:** 1 DDS, Graduate student, Department of Restorative Dentistry, Dental Materials Division, Piracicaba Dental School, State University of Campinas, Piracicaba, SP, Brazil.; 2 DDS, MS, Graduate student, Department of Restorative Dentistry, Dental Materials Division, Piracicaba Dental School, State University of Campinas, Piracicaba, SP, Brazil. Assistant Professor, Dental School, University of Uberaba, Uberaba, MG, Brazil.; 3 DDS, MS, PhD, Postdoctoral student, Department of Restorative Dentistry, Dental Materials Division, Piracicaba Dental School, State University of Campinas, Piracicaba, SP, Brazil.; 4 DDS, MS, PhD, Professor, Department of Restorative Dentistry, Dental School, Federal University of Pelotas, Pelotas, RS, Brazil.; 5 DDS, MS, PhD, Professor, Department of Restorative Dentistry, Dental Materials Division, Piracicaba Dental School, State University of Campinas, Piracicaba, SP, Brazil.

**Keywords:** Dental bonding, Dental cements, Dental porcelain, Tensile strength

## Abstract

**Objectives:**

The aim of this study was to investigate the bond strength of ceramic restorations luted using a self-adhesive resin cement (RelyX Unicem, 3M ESPE) under different dentin conditions.

**Material and Methods:**

In the experimental groups, ceramic restorations were luted to bovine incisors with RelyX Unicem under the following conditions: [Dry dentin]: surface was dried using air stream for 15 s; [Moist dentin]: excess dentin moisture was removed with absorbent paper; [Bonding agent]: Clearfil SE Bond (Kuraray) self-etching adhesive system was previously applied to dentin. In the Control group, cementation was done using an etch-and-rinse adhesive (Excite DSC) and Variolink II resin cement (Ivoclar Vivadent). Photoactivation of the resin cements was performed with UltraLume LED 5 unit (Ultradent). The restorations (n=5 per group) were sectioned into beams and microtensile testing was carried out. Data were subjected to ANOVA and Tukey's test (p<0.05). Failure modes were classified under Scanning Electron Microscopic (SEM) (×120 magnification).

**Results:**

The bond strength was dependent on the moisture status of the dentin. Bond strength in the "dry dentin group" was significantly lower than that of all other groups, which showed similar results. A predominance of mixed failures was detected for the control group, while a predominance of adhesive failures was observed for the "bonding agent" and "dry dentin" groups. The "moist dentin" group presented predominantly cohesive failures within the luting material. The previous application of a self-etching adhesive showed no significant effect.

**Conclusions:**

Only excess dentin moisture should be removed for the cementation of ceramic restorations with self-adhesive resin cements.

## INTRODUCTION

The luting procedure of ceramic restorations requires several sequential steps, and the use of adhesive systems associated with resin-based luting agents is very common^1,2,12^. In addition to etch-andrinse adhesives, self-etching systems are used with the purpose of eliminating the rinsing/drying steps and facilitating the bonding procedure. The self-etching approach also potentially reduces the occurrence of the postoperative sensitivity that may occur due to incomplete infiltration of the demineralized dentin^19^. Previous studies have reported similar bond strengths to dentin for some etch-and-rinse and self-etching systems depending on their composition and application steps^3,6,20^.

Self-adhesive luting materials were introduced in an endeavor to reduce the number of cementation steps by eliminating the previous application of bonding agent or other pre-treatment of the tooth^5,8,14-16^. The use of these materials should also prevent the incomplete infiltration of dentin and reduce the occurrence of postoperative sensitivity. Their adhesive properties are attributed to acidic monomers that simultaneously demineralize and infiltrate the tooth substrate, resulting in micromechanical retention. Secondary reactions have been suggested to provide additional chemical bonding to the dental hard tissues^7^.

The basic inorganic fillers in self-adhesive luting agents are able react with the phosphoric acid methacrylates present in the material^14^. The dominant setting reaction occurs via free radical polymerization, initiated either by light or a redox system that allows the polymerization in an acid environment^14^. Water has a critical role in bonding effectiveness: water is generated during neutralization of the functional groups modified by phosphoric acid and reused to react with acidic functional groups and ion-releasing basic filling bodies^14^. However, it is unknown whether the amount of water generated during cement setting is sufficient for proper bonding, or whether dentin moisture might influence the bonding mechanism.

The influence of previous application of a self-etching adhesive system on the bonding of self-adhesive luting agents is still unknown. Literature is lacking of studies evaluating the influence of dentin conditions on the performance of self-adhesive luting agent. The aim of this study was to investigate the bond strength to dentin of ceramic restorations luted with a self-adhesive resin luting agent under different dentin conditions: wet dentin, dry dentin or dentin previously treated with a self-etching adhesive. The null hypothesis tested was that substrate moisture and application of a self-etching system do not interfere with the bonding to dentin.

## MATERIAL AND METHODS

### Ceramic specimens

Rectangular specimens (10×8×2.5 mm) were made of leucite-reinforced glass ceramic (IPS empress esthetic; Ivoclar Vivadent, Schaan, Liechtenstein), shade eTC 2, used in accordance with the manufacturer’s instructions. Briefly, cylindrical patterns were made with organic wax, invested with phosphate-based material (esthetic Speed; Ivoclar Vivadent) and heated at 850ºC for 1 h in an oven (Vulcan A-550; Degussa-Ney, Yucaipa, CA, USA). The ceramic was then heat pressed into the molds, using the eP600 furnace (Ivoclar Vivadent). After cooling to room temperature, the specimens were divested, polished with 1200-grit SiC papers, and ultrasonically cleaned in water for 10 min. The internal surfaces of the ceramic blocks were etched with 10% hydrofluoric acid for 20 s, rinsed with water for 1 min, and received a layer of silane coupling agent (RelyX Ceramic Primer; 3M eSPe, St. Paul, MN, USA).

### Bonding procedures

Bovine incisors were obtained and their crowns were sectioned 7 mm below the incisal edge with a double-face diamond disc (#7020; KG Sorensen, São Paulo, SP, Brazil) under air-water cooling. The surrounding enamel was removed using diamond burs (#2214; KG Sorensen), the dentin surfaces were wet-polished with 600-grit SiC papers (Norton S.A., São Paulo, SP, Brazil), and the root portions of the teeth were embedded in epoxy resin. The teeth were randomly divided into four groups (n=5) defined by the dentin condition:

"Dry dentin" group: the dentin surface was dried with air for 15 s and the self-adhesive resin luting agent RelyX Unicem (3M eSPe, St. Paul, MN, USA), shade A2, was used following the manufacturer’s instructions;

"Moist dentin" group: only the excess dentin moisture was removed using absorbent paper, and the same procedures described for the previous group were performed;

"Bonding agent" group: the dentin surface was dried with air for 15 s and a two-step self-etching bonding agent (Clearfil Se Bond, Kuraray Co. Ltd., Osaka, Japan) was applied according to the manufacturer’s instructions, followed by application of RelyX Unicem, as described for the previous groups.

Control group: the dentin surface was dried with air for 15 s, etched with 37% phosphoric acid gel for 15 s, rinsed with water for 30 s, and blot dried leaving a moist surface. An etch-and-rinse adhesive system (excite DSC, Ivoclar Vivadent) and a dual-cured resin luting agent (Variolink II, Ivoclar Vivadent), shade A2, were used, according to the manufacturer instructions.

Figure 1 presents the composition of the materials used in the study. After applying the luting materials and positioning the ceramic blocks, the specimens were placed under a 500 g static load for 2 min, and the excess cement was removed with a disposable microbrush. Four 40-s light-activation exposures were performed at right angles using a LeD source (UltraLume LeD 5, Ultradent, South Jordan, UT, USA) 1200 mW/cm^2^), with a final 40-s exposure from the top surface.

**Figure 1:** Materials used in the study

**Material**	**Description**	**Manufacturer**	**Batch**	Main components *
IPS Empress Esthetic	Leucite-reinforced glass ceramic	Ivoclar Vivadent	JM0728	SiO2, BaO, Al2O3, CaO, CeO2, Na2O, K2O, B2O3, TiO2
RelyX Ceramic Primer	Silane coupling agent	3M ESPE	6XK	Methacryloxypropyl trimethoxysilane, ethanol, water
RelyX Unicem	Self-adhesive resin luting agent	3M ESPE	312491	Methacrylated phosphoric acid esters, TEGDMA, substituted dimethacrylate, glass/silica particles, calcium hydroxide, substituted pyrimidine, sodium persulfate
Variolink II	Dual-cured resin luting agent	Ivoclar Vivadent	Base: J19730 Catalyst: J21518	Bis-GMA, TEGDMA, UDMA, inorganic fillers, ytterbiumtrifluoride
Excite DSC	Two-step etch-and-rinse adhesive system	Ivoclar Vivadent	H02749	Dimethacrylates, alcohol, phosphonic acid acrylate, HEMA, silica particles
Clearfil SE Bond	Two-step self-etching adhesive system	Kuraray	C8039	10-MDP, hydrophobic and hydrophilic aliphatic dimethacrylates, water, colloidal silica

* Information provided by the manufacturers

### Bond strength testing

In order to obtain specimens for the microtensile test, blocks (4 mm in height) of self-polymerizing resin composite (Concise Orthodontics, 3M ESPE, St. Paul, MN, USA) were built-up on the ceramic surfaces to increase the height of the sample. The specimens were stored in 100% relative humidity at 37°C, for 24 h. Thereafter, the composite-ceramic-cement-tooth sets were cut perpendicular to the bonding interface into beam specimens using a water-cooled diamond saw (Isomet 1000, Buehler, Lake Bluff, IL, USA). The cross-sectional area of the bond interface of each beam was measured with a digital caliper (Mitutoyo Corporation, Tokyo, Japan) and the microtensile test conducted on a mechanical testing machine (Instron 4411, Instron Corp., Canton, MA, USA) at a crosshead speed of 0.5 mm/min until failure. Bond strength values were calculated in MPa. An average of six beams was obtained for each tooth, and the mean value of the six beams was computed as the bond strength value for each specimen. Bond strength data were subjected to one-way ANOVA and multiple comparisons were performed using the Tukey’s post-hoc test. Differences were considered significant at p<0.05. In the event of spontaneous debonding during the sectioning procedures, the specimens were excluded from the statistical analysis.

### Failure analysis

The fractured specimens were coated with gold and examined with a scanning electron microscope (SeM) (JSM5600LV, JEOL Inc., Peabody, MA, USA), at a ×120 magnification. Their modes of failure were classified using a modified criterion^10^ , as follows: adhesive failure (Mode 1), mixed failure involving bonding agent, dentin and luting material (Mode 2); mixed failure involving luting material and dentin (Mode 3); cohesive failure within the bonding agent (Mode 4); cohesive failure within the luting material (Mode 5).

## RESULTS

### Bond strength testing

Results for the microtensile bond strength test are shown in Table 1. The group in which the bonding was performed on dry dentin presented significantly lower bond strength compared with all remaining groups (p<0.01). The self-adhesive resin luting agent presented lower bond strength when applied to the dry compared with the moist dentin substrate (p<0.01). On the other hand, no significant differences were found when the moist dentin, bonding agent and control groups were compared with each other (p = 0.093).

**Table 1:** Means (standard deviations) for microtensile bond strength

**Group**	**Bond strength (MPa)**
	
Bonding agent	24.2 (2.6)**a**
Control	19.0 (5.0)**a**
Moist dentin	18.5 (3.2)**a**
Dry dentin	9.1 (2.8)**b**

Different letters indicate statistically significant differences (Tukey's test, p<0.05).

### Failure analysis

**Figure 2:**
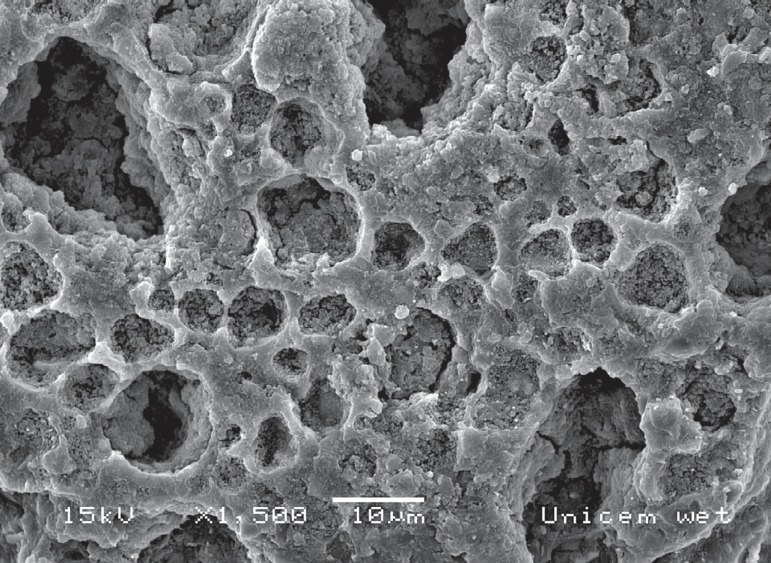
Representative Scanning Electron Microscope (SEM) micrograph of a cohesive failure within the self-adhesive resin cement in the "moist dentin" group. Porosity can be observed in the bulk of the luting agent, probably resulting from oversaturated water droplets accumulating in microvoids within the polymer network, decreasing its cohesive strength

The failure analysis demonstrated that the mode 2 was the predominant mode of failure for the control group. The bonding agent and the dry dentin groups showed a predominance of failure mode 1. In contrast, a predominance of failure mode 5 was detected for the group in which the bonding was performed to moist dentin. The percentage of failure modes in each group is shown in Table 2. Figure 2 shows a representative SeM image of a cohesive failure of the "moist dentin" group: porosity was observed into the bulk of the luting agent.

**Table 2:** Scanning Electron Microscope (SEM) classification of the failure modes

**Group**	**Mode 1**	**Mode 2**	**Mode 3**	**Mode 4**	**Mode 5**
					
Bonding agent	45%	25%	-	5%	25%
Control	25%	55%	-	5%	15%
Moist dentin	-	-	10%	-	90%
Dry dentin	80%	-	-	-	20%

Failure classification: Mode 1: adhesive failure; Mode 2: mixed failure involving bonding agent, dentin and luting material; Mode 3: mixed failure involving luting material and dentin; Mode 4: cohesive failure within the bonding agent; Mode 5: cohesive failure within the luting material

## DISCUSSION

The null hypothesis tested in this study was rejected, as the self-adhesive cement had lower bond strength to the dry compared with the moist dentin. RelyX Unicem needs water for ionization of the acidic monomers to modify the smear layer and interact with the dentin. The initially anhydrous cement bonds to the substrate via mechanisms of water generation and subsequent water recycling, as proposed by the manufacturer^14^. However, the current results suggest that the water present in the substrate might also play an important role on the bonding mechanism. The increased water availability on the dentin probably improved the acid ionization and etching effect, enhancing the bond between the negatively charged phosphoric acid groups to the Ca ions on dentin. This result is in line with a recent study^9^ , which observed increased bond strength to dentin when a self-adhesive cement was applied under simulated pulpal pressure.

Adhesive systems promote better interaction with the dentin than self-adhesive cements, due to the infiltration of the bonding agent into the substrate and formation of a hybrid layer^5,17^. Although previous studies have suggested that the weak link in self-adhesive luting systems lies in their lack of genuine hybridization of the bonding surfaces^4,13^ , similar bond strengths were observed for the "moist dentin" group compared to the "bonding agent" and control groups in the present study. Therefore, it seems that application of a self-etching adhesive prior to the use of the self-adhesive luting agent has no beneficial effect for self-adhesive cements. Nonetheless, it is difficult to predict whether similar long-term performances would be observed among these groups, as the quality of the hybrid layer formed is related to the resistance to bond degradation over the course of time^11^.

On the other hand, different failure results were detected among the groups. A predominance of adhesive failures was observed for the dry substrate, confirming the weak interaction between the self-adhesive cement and dry dentin surface. This can be explained by the lower water availability, poorer ionization and, in association with the high viscosity of the cement, insufficient monomer infiltration into the substrate. In contrast, cohesive failures within the luting agent were predominant in the "moist dentin" group. This result might suggest that the mechanism of bonding to moist dentin was improved. However, as shown in Figure 1, porosity was observed in the bulk of the luting agent, probably resulting from oversaturated water droplets accumulating in microvoids within the polymer network, decreasing its cohesive strength.

In the control group, there was a predominance of mixed failures involving bonding agent, dentin and luting material. This might be explained by the in-depth demineralization of the dentin by the phosphoric acid, leaving non-encapsulated collagen fibrils after bonding, because of the inability of the bonding agent to fully infiltrate the exposed mesh^18^. These unprotected areas may have served as spots for stress concentration during the tensile test, generating failures involving not only the bonding layer, but also the dentin tissue. In contrast, the predominance of adhesive failures for the self-etching system is probably related to its lower ability in creating micromechanical retention compared to the etch-andrinse adhesive has, leading to failures mainly at the dentin-adhesive interface.

The present study has clinical implications. Although in dental practice it is difficult to control the state of hydration of dentin for proper bonding, it is advisable to use absorbent paper only to remove the excess water and not to over-dry the dentin surface when using self-adhesive luting agents. However, the conditions of this *in vitro* study do not take into account the effect that the pulpal pressure might have on dentin permeability^9^ , which could potentially overcome the lower water availability. In addition, it is uncertain whether the previous application of bonding agent could affect the polymer network formation of the cement. Moreover, the long-term bonding performance of the materials and techniques tested in the present study must be investigated. Therefore, further laboratory and clinical studies are necessary.

## CONCLUSION

The bond strength of the self-adhesive luting agent RelyX Unicem was dependent mainly on the moisture status of the dentin. The findings of this study indicate that only the excess dentin moisture should be removed during cementation of ceramic restorations using self-adhesive resin cements.

## References

[1] Chaves CAL, Melo RM, Passos SP, Camargo FP, Bottino MA, Balducci I (2009). Bond strength durability of self-etching adhesives and resin cements
to dentin. J Appl Oral Sci.

[2] Della-Bona A (2005). Characterizing ceramics and the interfacial adhesion to resin: II- the
relationship of surface treatment, bond strength, interfacial toughness and
fractography. J Appl Oral Sci.

[3] De Munck J, Shirai K, Yoshida Y, Inoue S, Van Landuyt K, Lambrechts P (2006). Effect of water storage on the bonding effectiveness of 6 adhesives to
Class I cavity dentin. Oper Dent.

[4] De Munck J, Vargas M, Van Landuyt K, Hikita K, Lambrechts P, Van Meerbeek B (2004). Bonding of an auto-adhesive luting material to enamel and
dentin. Dent Mater.

[5] Frankenberger R, Lohbauer U, Schaible RB, Nikolaenko SA, Naumann M (2008). Luting of ceramic inlays in vitro: marginal quality of self-etch and
etch-and-rinse adhesives versus self-etch cements. Dent Mater.

[6] Frankenberger R, Tay FR (2005). Self-etch vs etch-and-rinse adhesives: effect of thermo-mechanical
fatigue loading on marginal quality of bonded resin composite
restorations. Dent Mater.

[7] Gerth HU, Dammaschke T, Züchner H, Schäfer E (2006). Chemical analysis and bonding reaction of RelyX Unicem and Bifix
composites -a comparative study. Dent Mater.

[8] Han L, Okamoto A, Fukushima M, Okiji T (2007). Evaluation of physical properties and surface degradation of
self-adhesive resin cements. Dent Mater J.

[9] Mazzitelli C, Monticelli F, Osorio R, Casucci A, Toledano M, Ferrari M (2008). Effect of simulated pulpal pressure on self-adhesive cements bonding
to dentin. Dent Mater.

[10] Moraes RR, Gonçalves LS, Ogliari FA, Piva E, Sinhoreti MA, Correr-Sobrinho L (2008). Development of dental resin luting agents based on BisEMA4: bond
strength evaluation. Express Polym Lett.

[11] Okuda M, Pereira PN, Nakajima M, Tagami J, Pashley DH (2002). Long- term durability of resin dentin interface: nanoleakage vs.
microtensile bond strength. Oper Dent.

[12] Piva E, Correr-Sobrinho L, Sinhoreti MA, Consani S, Demarco FF, Powers JM (2008). Influence of energy density of different light sources on Knoop
hardness of a dual-cured resin cement. J Appl Oral Sci.

[13] Piwowarczyk A, Bender R, Ottl P, Lauer HC (2007). Long-term bond between dual-polymerizing cementing agents and human
hard dental tissue. Dent Mater.

[14] RelyX(tm) Unicem self-adhesive universal resin cement - technical product
profile.

[15] Saskalauskaite E, Tam LE, McComb D (2008). Flexural strength, elastic modulus, and pH profile of self-etch resin
luting cements. J Prosthodont.

[16] Senyilmaz DP, Palin WM, Shortall AC, Burke FJ (2007). The effect of surface preparation and luting agent on bond strength to
a zirconium- based ceramic. Oper Dent.

[17] Torres CRG, Pinto LQ, Leonel AG, Pucci CR, Borges AB (2007). Interaction between total-etch and self-etch adhesives and
conventional and self-adhesive resin cements. Braz J Oral Sci.

[18] Wang Y, Spencer P (2005). Evaluation of the interface between one-bottle adhesive systems and
dentin by Goldner's trichrome. Am J Dent.

[19] Wang Y, Spencer P (2003). Hybridization efficiency of the adhesive/dentin interface with wet
bonding. J Dent Res.

[20] Yesilyurt C, Bulucu B (2006). Bond strength of total-etch and self-etch dentin adhesive systems on
peripheral and central dentinal tissue: a microtensile bond strength
test. J Contemp Dent Pract.

